# Trastuzumab emtansine versus trastuzumab plus pertuzumab for HER2-positive breast cancer with residual disease after neoadjuvant therapy: a real-world study

**DOI:** 10.3389/fonc.2026.1806399

**Published:** 2026-06-18

**Authors:** Zhong Wang, Yimin Zhang, Feng Yao, Jinxin Li, Chenyuan Li, Shichong Liao

**Affiliations:** 1 Department of Breast and Thyroid Surgery, Renmin Hospital of Wuhan University, Wuhan, Hubei, China; 2 Department of Academic Affairs, School of Medicine, Wuhan University, Wuhan, Hubei, China

**Keywords:** disease-free survival, early breast cancer, HER2-positive, non-pCR, trastuzumab emtansine

## Abstract

**Objective:**

To evaluate the efficacy and safety of T-DM1 versus trastuzumab plus pertuzumab in adjuvant treatment of HER2-positive breast cancer patients with residual invasive disease after neoadjuvant therapy.

**Methods:**

This study retrospectively analyzed 184 patients treated at Renmin Hospital of Wuhan University from January 2019 to January 2022. The patients were divided into a T-DM1 treatment group and a dual-target treatment group based on whether they received T-DM1 or trastuzumab plus pertuzumab during adjuvant therapy. Clinical and pathological data of the two groups were collected before and after surgery, and follow-up was conducted. The primary study endpoint was disease-free survival (DFS).

**Results:**

Disease progression occurred in 20.3% of the dual-target treatment group and 12.6% of the T-DM1 treatment group. Although T-DM1 was associated with a 38% reduction in recurrence risk, the difference was not statistically significant (HR: 0.62, 95% CI: 0.30-1.26, P = 0.19). Exploratory subgroup analyses suggested improved DFS with T-DM1 in patients with initially unresectable disease, preoperative lymph node positivity, or poor pathological response (MP 1–3 or RCB III); In terms of safety, both groups of patients were consistent with the known safety profile. No treatment-related deaths occurred. Grade ≥3 thrombocytopenia was significantly more frequent with T-DM1 (8.45% vs 0.88%, P = 0.01), representing a clinically important toxicity trade-off.

**Conclusion:**

The use of T-DM1 in the adjuvant setting suggests improved disease-free survival in HER2-positive non-pCR patients who were initially inoperable, had preoperative lymph node positivity, or exhibited poor pathological response (MP grade 1–3 or RCB III).

## Introduction

In patients with HER2-positive early breast cancer, residual invasive disease after neoadjuvant therapy is associated with higher recurrence and mortality compared with pathological complete response (pCR) (1–3). The KATHERINE trial demonstrated that adjuvant T−DM1 improves invasive disease−free survival (iDFS) compared with trastuzumab alone in patients without pCR. At a median follow−up of 8.4 years, the 7−year iDFS rate was 80.8% with T−DM1 versus 67.1% with trastuzumab, with consistent benefits across all subgroups receiving T−DM1 intensification. The 7−year overall survival rate was 89.1% versus 84.4%, representing an absolute survival gain of 4.7% (HR: 0.66, 95% CI 0.51–0.87, P = 0.0027). Safety findings were favorable, with few adverse events, no new safety signals in patients completing 14 cycles of T−DM1, and a low incidence of cardiac toxicity (4). The KATHERINE trial remains the landmark study establishing the benefit of T−DM1 for non−pCR patients after neoadjuvant therapy for early breast cancer, and it has profoundly shaped contemporary clinical practice.

However, evolving treatment paradigms limit the generalizability of the KATHERINE findings to contemporary HER2-positive breast cancer care. Trials including NeoSphere (5) and PEONY (6, 7) have further validated the efficacy and safety of neoadjuvant trastuzumab plus pertuzumab, substantially influencing clinical practice. Accordingly, current global guidelines recommend dual-target therapy as standard neoadjuvant treatment for HER2-positive early breast cancer. Notably, fewer than 20% of patients in KATHERINE received early dual-target therapy, and non-pCR patients in that trial were treated with adjuvant trastuzumab monotherapy (4). Meanwhile, the APHINITY trial demonstrated that adjuvant dual-target therapy significantly improves iDFS, particularly in lymph node-positive patients (HR = 0.72, 95% CI 0.60–0.87), establishing it as a new standard of care for high-risk disease (8, 9). Critically, KATHERINE only compared T-DM1 with trastuzumab monotherapy in the adjuvant setting, leaving unanswered whether T-DM1 or continued dual-target therapy yields superior adjuvant efficacy. This study therefore aims to compare the efficacy and safety of adjuvant dual-target therapy versus T-DM1 in non-pCR patients who received neoadjuvant dual-target therapy.

## Methods

### Study population

This retrospective study enrolled patients with breast cancer who received neoadjuvant therapy at the Breast and Thyroid Center of Wuhan University People’s Hospital between January 2019 and January 2022. Eligible patients had histologically confirmed HER2-positive early invasive breast cancer, received at least 12 weeks of neoadjuvant trastuzumab plus pertuzumab (dual-target therapy), and had non-pathological complete response (non-pCR) confirmed by surgical pathology. Exclusion criteria included severe cardiopulmonary dysfunction, incomplete clinicopathological data, or loss to follow-up. A total of 184 patients were included. Treatment regimens are formulated by physicians on a case-by-case basis in accordance with institutional clinical protocols and clinical practice guidelines. T-DM1 (trastuzumab emtansine) is preferentially recommended for patients with initially unresectable disease, high nodal burden (clinical stage N2–N3), or poor pathological response (MP grading 1–3). Patients with low residual disease burden or intolerance to T-DM1 may receive dual-target maintenance therapy. Decisions also take into account patient preferences, economic accessibility, and drug availability. According to the different treatments used by patients in the real world during the postoperative adjuvant treatment phase, such as dual-target therapy and T-DM1 therapy, the patients were divided into a dual-target therapy group and a T-DM1 therapy group. There were 113 cases in the dual-target therapy group and 71 cases in the T-DM1 therapy group.

### Research methods

Postoperative adjuvant therapy should be started within 12 weeks after surgery. For trastuzumab, a loading dose of 8 mg/kg was administered if more than 6 weeks had elapsed since the last dose; otherwise, a maintenance dose of 6 mg/kg was given every 3 weeks. For pertuzumab, a loading dose of 840 mg was administered if the interval exceeded 6 weeks, followed by a maintenance dose of 420 mg every 3 weeks. The dose of T-DM1 is 3.6mg/kg, and if drug-related adverse reactions occur during treatment, the drug dose will be adjusted according to the drug instructions. The adjuvant treatment phase requires completion of 14 treatments. During the adjuvant treatment period, other breast cancer treatments such as radiotherapy or endocrine therapy are given according to guidelines or routine practices.

### Data collection and follow-up

The collected clinical pathological data include: age, gender, ethnicity, pre- and postoperative tumor size, lymph node metastasis, liver and renal function, blood routine, echocardiography, ER, PR status, Miller-Payne(MP) grading(Ranges from Grade 1:no change or minimal change in malignant cells, to Grade 5:no invasive or *in situ* cancer cells remaining), Residual Cancer Burden(RCB) grading, and other clinical pathological data. All patients were followed up until May 2024. The main collections during follow-up included the patients’ disease-free survival, overall survival, and sites of recurrence and metastasis.

### Statistical analysis

All analyses were performed using SPSS 22.0. Continuous variables are presented as mean ± standard deviation and compared using the t-test; categorical variables are summarized as frequencies and percentages and compared using the chi-square test or Fisher’s exact test, as appropriate. Unadjusted Cox proportional hazards models were used to estimate hazard ratios (HR) and 95% confidence intervals (CI) for DFS and OS between groups. Due to the limited sample size, multivariable adjustment was not performed. No formal testing of the proportional hazards (PH) assumption was conducted; HRs are therefore interpreted as average effects over follow-up. DFS was defined as the time from surgery to the first invasive recurrence (local, regional, or distant), contralateral invasive breast cancer, or death from any cause, whichever occurred first. OS was defined as the time from surgery to death from any cause. Patients without an event were censored at the last follow-up date (May 2024). Median follow-up was calculated using the reverse Kaplan–Meier method. Survival curves were generated using the Kaplan–Meier method and compared with the log-rank test. A two-sided P-value < 0.05 was considered statistically.

## Results

### Population

Among the 184 patients included in this study, there were 113 patients in the dual-target therapy group and 71 patients in the T-DM1 therapy group. The average follow-up time for the dual-target therapy group was 40.24 ± 11.84 months, while for the T-DM1 group it was 40.63 ± 7.80 months. Baseline demographics and clinicopathological characteristics were generally balanced between the two groups (**Table 1**): Overall, 69.02% of patients were hormone receptor-positive, 85% received the TCbHP neoadjuvant regimen, and 66.84% had preoperative axillary lymph node metastasis.

**Table 1:** Demographic and clinical characteristics of the patients at baseline.

Indicators	Dual target therapy	TDM1	P
Average Age(Y)	54.43 ± 10.65	52.06 ± 8.77	0.36
Gender			0.39
Female	100%	98.59%	
Male	0	1.41%	
Ethnicity			0.99
Han Chinese	97.35%	97.18%	
Other	2.65%	2.82%	
Clinical Stage			0.75
Operable	68.14%	71.23%	
Inoperable	31.86%	28.77%	
Hormone Receptor Status			0.75
ER or PR positive	69.91%	67.12%	
ER and PR negative	30.09%	32.88%	
Preoperative Neoadjuvant Regimen			0.55
TCbHP	94	63	
EC-THP	8	4	
THP	11	4	
T stage			0.94
1	12	6	
2	57	36	
3	30	21	
4	14	8	
N stage			0.19
0	40	21	
1	38	35	
2	30	13	
3	5	2	
Median Follow-up Time (months)	40.24 ± 11.84(16-63)	40.63 ± 7.80(19-58)	0.31

### Efficacy

**Figure 1:**
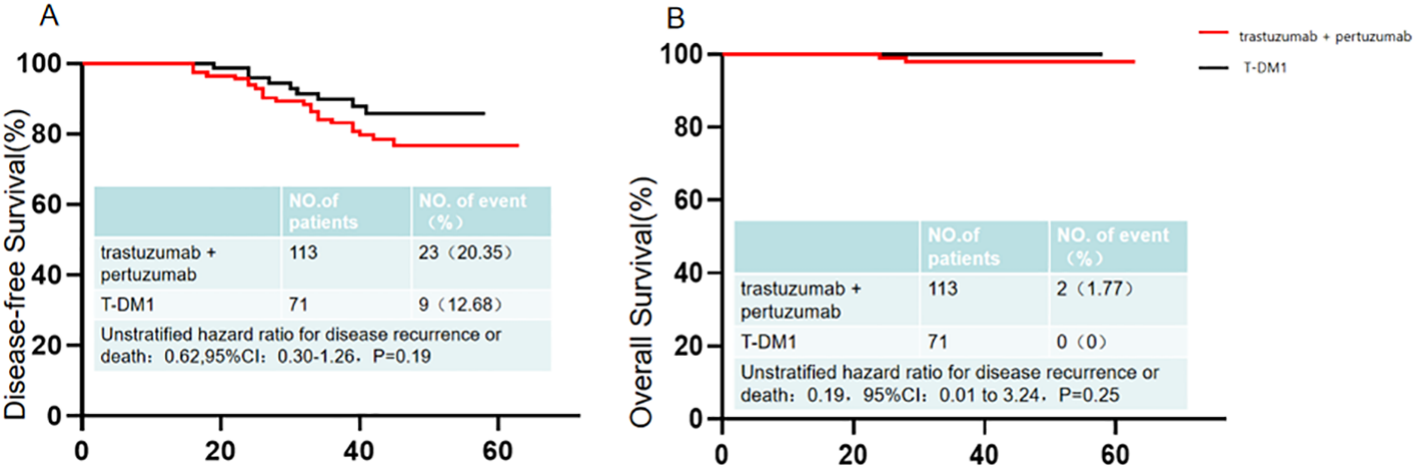
Kaplan–Meier estimates of survival. **(A)** Disease-free Survival was defined as the time from randomization until the date of the first occurrence of one of the following: recurrence of ipsilateral invasive breast tumor, recurrence of ipsilateral locoregional invasive breast cancer, contralateral invasive breast cancer, a distant disease recurrence, or death from any cause. **(B)** Overall Survival: From the start of treatment to death from any cause.

Follow-up was completed in May 2024. Median follow-up was 40.2 months (dual-target) and 40.6 months (T-DM1). Events occurred in 23/113 (20.35%) of the dual-target group and 9/71 (12.68%) of the T-DM1 group; all other patients were censored at last contact. Unadjusted Cox regression showed a non-significant reduction in recurrence risk with T-DM1 (HR = 0.62, 95% CI: 0.30–1.26, P = 0.19). (**Figure 1A**). As of May 2024, no deaths have occurred in the T-DM1 treatment group, while there were 2 deaths in the dual-target treatment group (HR: 0.19, 95% CI: 0.01 to 3.24, P = 0.25)(**Figure 1B**).

**Figure 2:**
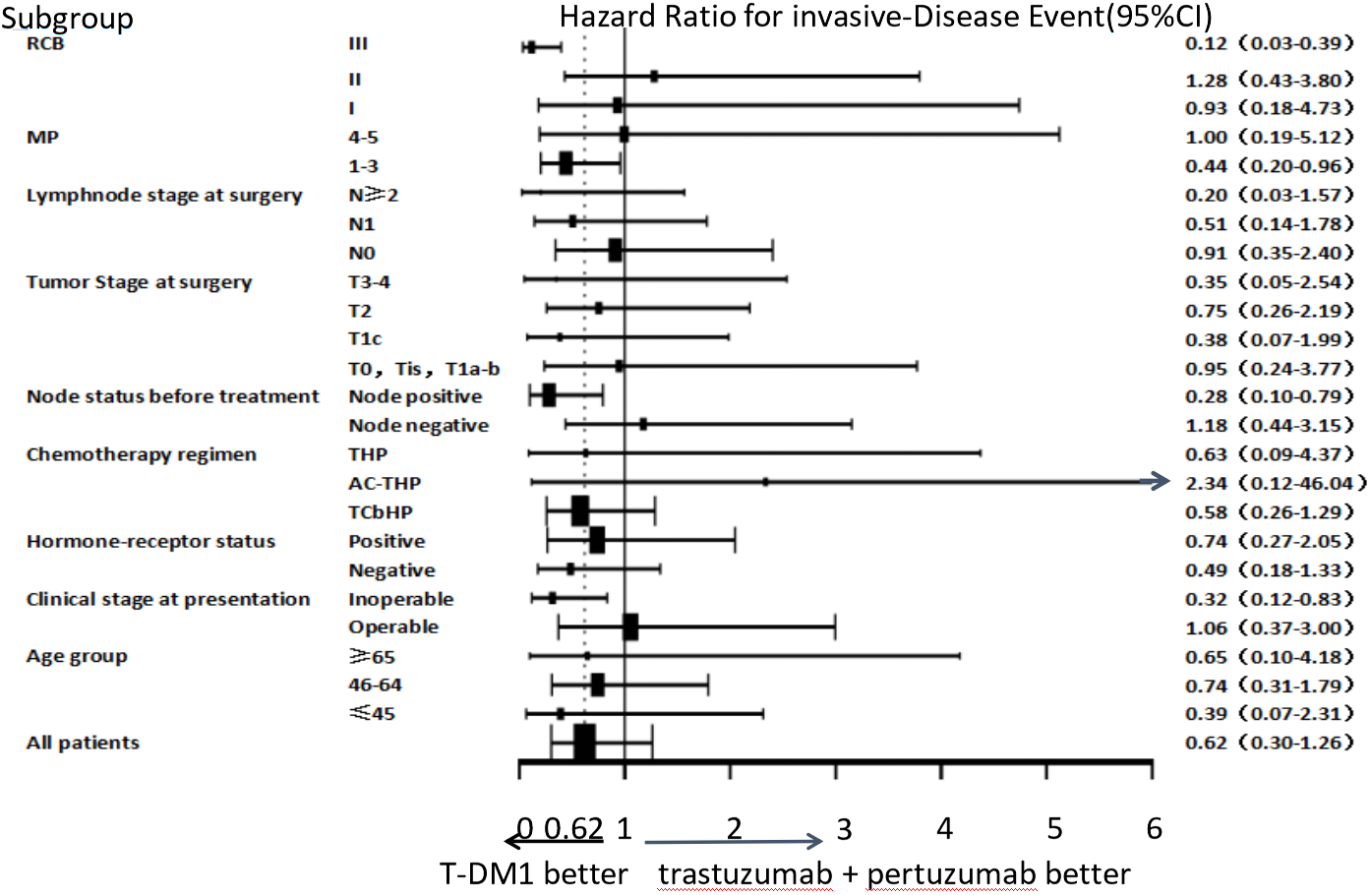
Exploratory subgroup analysis of disease–free survival.

In exploratory subgroup analyses for DFS, no significant between-group differences were observed according to age, hormone receptor status, neoadjuvant regimen, or postoperative T and N stages. However, exploratory analyses showed that adjuvant T-DM1 significantly improved DFS in patients with initially unresectable disease (T4Nx or TxN2–3) (HR = 0.32, 95% CI: 0.12–0.83, P = 0.04). Similarly, T-DM1 was associated with better DFS in preoperative lymph node-positive patients (HR = 0.28, 95% CI: 0.10–0.79, P = 0.02). Improved DFS with T-DM1 was also seen in patients with poor pathological response to neoadjuvant therapy, including those with MP grades 1–3 (HR = 0.44, 95% CI: 0.20–0.96, P = 0.04) or RCB III disease (HR = 0.12, 95% CI: 0.03–0.39, P = 0.01) (**Figure 2**).

### Safety

The incidence of treatment-related adverse events was 90.27% in the dual-target group versus 95.77% in the T-DM1 group (P = 0.25). Diarrhea was the most common adverse event with dual-target therapy (38.93%), whereas fatigue predominated with T-DM1 (71.83%). The overall rate of grade ≥3 adverse events was numerically higher with T-DM1 (15.49% vs. 13.27%, P = 0.67), with few grade 4 events and no treatment-related deaths in either group. Treatment delays or dose adjustments due to toxicity were more frequent with T-DM1 (8.45% vs. 4.42%), though the difference was not statistically significant. Both groups completed a median of 14 cycles (range: 1–14).Notably, the safety profiles differed substantially in clinically meaningful ways.​ Grade ≥3 adverse events in the T-DM1 group were dominated by severe thrombocytopenia (8.45%) and fatigue (7.04%). Critically, grade 3–4 thrombocytopenia was the primary driver of dose modifications and treatment interruptions,​ leading to permanent discontinuation in 2 patients (2.82%). By contrast, severe toxicity in the dual-target group consisted mainly of diarrhea and fatigue (both 5.31%). While the overall frequency of grade ≥3 events was similar (P = 0.67), the qualitative nature of these toxicities reflects a distinct clinical trade-off:​ T-DM1 confers a significantly higher risk of hematologic toxicity requiring intensive monitoring, whereas dual-target therapy is characterized by manageable gastrointestinal events. No patients died from treatment-related toxicity (**Table 2**).

**Table 2:** Summary of adverse events in the safety population.

Adverse events	Dual target therapy	T-DM1	P
Median cycles completed	14	14	>0.99
Any adverse events	90.27%	95.77%	0.25
Adverse events of grade 3 or above	13.27%	15.49%	0.67
Adverse events leading to death	0%	0%	
Severe adverse events (Grade IV)	1.77%	2.82%	0.64
Permanent discontinuation	0.88%	2.82%	0.39
Adverse events leading to treatment delay or dose adjustment	4.42%	8.45%	0.34
Grade≥3 adverse events that occurred in more than 1% of patients in either group			
Fatigue	5.31%	7.04%	0.75
Nausea and vomiting	1.77%	4.23%	0.37
Platelet decline	0.88%	8.45%	0.01
Neutrophil decline	1.77%	2.82%	0.64
Diarrhea	5.31%	1.41%	0.25
Peripheral neuriti	0%	1.41%	0.39

## Discussion

Currently, the use of T-DM1 for intensified treatment during the adjuvant therapy period for HER2-positive non-pCR early breast cancer patients who have undergone neoadjuvant therapy has been recommended by major guidelines (10–12). This is mainly based on the main conclusions of the Katherine study. However, the Katherine study was initiated earlier, when neoadjuvant dual−target therapy was not widely used. Moreover, the control group during the adjuvant period did not receive dual-target drug treatment, which differs significantly from current clinical practice. To clarify these issues, we retrospectively analyzed 184 HER2-positive non-pCR patients who received neoadjuvant dual-target therapy, stratifying them into adjuvant dual-target and T-DM1 groups. The results showed that although the use of T-DM1 in the overall population can reduce the risk of recurrence by 38%, the difference was not statistically significant. No significant difference in mortality risk was observed. Exploratory subgroup analysis showed that regardless of the patient’s age, preoperative neoadjuvant treatment regimen, hormone receptor status, postoperative T stage, N stage, there was no significant difference in DFS between the T-DM1 treatment group and the dual-target treatment group. However, T-DM1 can significantly improve the DFS of patients with initially unresectable disease, had lymph node metastasis before surgery, or had poor efficacy in neoadjuvant treatment, such as patients with MP grading of 1–3 or RCBIII (P values were 0.04, 0.02, respectively). In terms of adverse reactions, These safety findings represent a clinically important trade-off that should guide treatment decisions.​ Consistent with prior reports, T-DM1 demonstrated a distinct toxicity profile characterized by substantial fatigue and a significant risk of severe thrombocytopenia, particularly in the Asian population where the incidence of grade 3 thrombocytopenia (8.45%) is notably higher than that seen with dual-target therapy (0.88%, P = 0.01).While dual-target therapy is primarily associated with manageable gastrointestinal toxicity (diarrhea), T-DM1 imposes a different burden, with over 70% of patients experiencing fatigue and nearly 1 in 12 patients facing grade 3 cytopenias.​ Therefore, the choice of adjuvant therapy should be individualized. For patients with initially inoperable disease or high residual burden (MP 1-3/RCB III), the potential DFS benefit may justify the intensified monitoring required for T-DM1-related toxicities. Conversely, for lower-risk non-pCR patients, the dual-target regimen offers a favorable alternative with a different, often more tolerable, side effect profile.

The present study found that the mean follow-up duration was 40.63 months in the T-DM1 group, with a DFS rate of 85.78%, versus 40.24 months and 76.83% in the dual-target group. These figures are numerically lower than the 3-year DFS rates of 88.3% (T-DM1) and 77% (trastuzumab) reported in the KATHERINE trial. Possible explanations include the more advanced disease stage and higher proportion of initially inoperable patients in our cohort. Additionally, although neoadjuvant dual-target therapy is more intensive than single-target treatment, many patients still failed to achieve pCR, which may reflect inherently more aggressive tumor biology or reduced sensitivity to targeted therapy in this population. According to the latest data from the Katherine study, the 7-year iDFS was 80.8% vs. 67.1%, and benefits were obtained in all subgroups. The absolute difference in 7-year OS between T-DM1 and dual-target also increased by 4.7%, P = 0.0027. This indicates that in non-pCR patients, the use of T-DM1 in adjuvant treatment is superior to trastuzumab monotherapy. In the APHINITY study, during adjuvant treatment, the use of trastuzumab + pertuzumab compared to trastuzumab alone, with a median follow-up of 8.4 years, it was found that in the ITT population, the dual-target treatment group could reduce the relative risk of recurrence by 23% (HR 0.77, 95% CI: 0.66-0.91), and the difference was even more significant in the lymph node-positive group (HR 0.72, 95%CI: 0.60-0.87). Both of these clinical studies indicate that dual-target therapy or T-DM1 has superior efficacy to trastuzumab monotherapy during the adjuvant treatment period. However, the application scenarios of the two are not comparable, so the results of this study are more valuable. The results of this study indicate that not all non-pCR patients need to use T-DM1 for intensified treatment. Considering the higher incidence of adverse drug reactions with T-DM1, it is only necessary to consider using T-DM1 for intensified treatment in patients who are inoperable before surgery, have lymph node metastasis, or have poor efficacy in neoadjuvant treatment, such as patients with MP grading of 1–3 or RCBIII. In other cases, it is possible to continue using dual-target treatment, which can ensure efficacy and reduce the probability of toxic side effects.

Similar to this study, multiple analyses of T-DM1 clinical trials have observed an increased incidence of T-DM1-related thrombocytopenia in the Asian population (13, 14). In a combined analysis of seven studies, the incidence of grade 3–4 thrombocytopenia in the Asian population was 44%, while the incidence of thrombocytopenia in the non-Asian population was only 10.6% (13). Moreover, in the Katherine study, most patients with thrombocytopenia could recover to grade 1 and continue treatment, and more than 90% of the Chinese population could complete 7 cycles of T-DM1, but only 62.7% of the Chinese population could complete 14 cycles (4). Current research suggests that platelet counts should be tested before starting T-DM1 and before each dose. The potential mechanism underlying the increased incidence of thrombocytopenia in the Asian population is unclear. It is currently believed that T-DM1 is internalized by macrophages in a FC receptor-dependent manner and inhibits further differentiation of macrophages, leading to thrombocytopenia. A more common FC receptor-specific polymorphism in the Asian population is currently considered a potential mechanism (13, 15–17).

Although this study provides preliminary evidence for adjuvant treatment selection in HER2-positive patients with non-pCR, several limitations should be acknowledged. First, the lack of a statistically significant difference in the overall population (HR: 0.62, 95% CI: 0.30–1.26, P = 0.19) warrants cautious interpretation and cannot be directly regarded as evidence of therapeutic equivalence. The data suggest a clinically relevant trend favoring T-DM1, which likely failed to reach statistical significance due to insufficient statistical power resulting from the limited sample size. Second, this is a single-center retrospective study. While baseline characteristics were balanced, residual confounding cannot be excluded. Therefore, prospective, multicenter, randomized controlled trials (RCTs)​ are required to validate the efficacy and safety of T-DM1 versus dual-target therapy. Third, the study spanned a long period, and the continuous evolution of treatment paradigms may have influenced key endpoints such as DFS and OS, potentially introducing bias. Finally, the current follow-up duration is relatively short, with a limited number of DFS events. Longer follow-up may alter the observed outcomes.

In summary, although T-DM1 was associated with a numerically lower recurrence risk (38% reduction) in HER2-positive non-pCR patients, the primary endpoint was not statistically significant in the overall population. Exploratory subgroup analysis shows that the use of T-DM1 can improve the DFS of patients with initially inoperable disease, preoperative lymph node positivity, or poor neoadjuvant pathological response. This provides new insights for the adjuvant treatment of HER2-positive non-pCR patients after neoadjuvant treatment. However, these findings are hypothesis-generating and require validation in large-scale prospective trials.

## Data Availability

The original contributions presented in the study are included in the article/supplementary material. Further inquiries can be directed to the corresponding author.
